# Pharmacogenomics of surgical stress response in elective abdominal surgeries

**DOI:** 10.6026/973206300214005

**Published:** 2025-11-15

**Authors:** Tirth Hareshkumar Vyas, Mahi Jayeshbhai Khiloshiya, Nisha Jayantilal Parmar, Shreya Girishbhai Kaneria, Megha Bhaumik Patel

**Affiliations:** 1 Department of Medicine, B.J. Medical College, Ahmedabad, Gujarat, India; 2 Department of Casualty, GMERS Medical College and Civil Hospital, Junagadh, Gujarat, India; 3 Department of Pathology, Dr. N.D. Desai Faculty of Medical Science and Research, Dharmsinh Desai University, Nadiad, Gujarat, India; 4 Department of Medicine, GMERS Medical College and Civil Hospital, Junagadh, Gujarat, India; 5 Department of Public Health, Long Island University, USA

**Keywords:** Pharmacogenomics, surgical stress response, abdominal surgery, CYP2D6, OPRM1, personalized medicine, perioperative care

## Abstract

The variability in surgical stress response among patients undergoing elective abdominal surgeries remains inadequately understood, particularly in relation to genetic polymorphisms influencing drug metabolism and pain modulation. Hence, this study explored the association between key pharmacogenetic variants (CYP2D6, CYP3A4, OPRM1 and COMT) and biomarkers of surgical stress response. A total of 240 patients were evaluated, with half receiving pharmacogenomic-guided perioperative management. Significant differences were observed in IL-6 levels, opioid requirements and complication rates between genotypic groups. Pharmacogenomic-guided therapy effectively reduced postoperative complications and improved recovery, underscoring its potential for personalized surgical care.

## Background:

The field of perioperative medicine has witnessed remarkable advances in recent years, with increasing recognition of the importance of personalized approaches to surgical care [1]. Among these approaches, pharmacogenomics has emerged as a critical component in understanding individual variations in drug response and optimizing perioperative management [2]. The surgical stress response, characterized by complex neuroendocrine and inflammatory cascades, represents a significant determinant of postoperative outcomes and recovery trajectories [3]. Elective abdominal surgeries encompass a wide range of procedures that induce substantial physiological stress, triggering the release of cytokines, hormones and acute-phase proteins [4]. This stress response, while essential for healing, can become maladaptive and contribute to postoperative complications, prolonged hospitalization and increased healthcare costs [5]. Recent studies have demonstrated that genetic factors play a crucial role in modulating both the intensity of surgical stress response and individual responses to perioperative medications [6]. Enhanced Recovery After Surgery (ERAS) protocols have transformed perioperative management by emphasizing a multimodal and multidisciplinary approach to optimize surgical outcomes and shorten recovery time. Comprehensive reviews highlight that anaesthetic management plays a crucial role within ERAS pathways by minimizing surgical stress responses and facilitating faster postoperative rehabilitation [7]. Clinical evaluations of ERAS implementation in laparoscopic procedures, such as living donor nephrectomy, have shown reductions in hospital stay duration and postoperative pain while maintaining patient safety and comfort [8]. Similarly, randomized controlled trials in laparoscopic liver resections have demonstrated that ERAS protocols significantly decrease complication rates, hospital costs, and recovery time when compared with conventional perioperative care [9]. Recent advances in genomic technologies have made pharmacogenetic testing more accessible and cost-effective, paving the way for its integration into routine clinical practice [10]. Several studies have demonstrated the potential benefits of pharmacogenomic-guided therapy in reducing adverse drug events and improving outcomes in various clinical settings [11]. However, the application of pharmacogenomics specifically in the context of surgical stress response remains relatively unexplored, representing a significant research gap [12]. The relationship between genetic polymorphisms and surgical stress biomarkers has been investigated in limited studies, with preliminary evidence suggesting that certain genetic variants may predispose patients to exaggerated inflammatory responses and poorer outcomes [13]. Recent evidence supports the effectiveness of Enhanced Recovery After Surgery (ERAS) protocols beyond traditional abdominal and thoracic procedures, extending their benefits to neurosurgical interventions as well. The study by Wang et al. demonstrated that applying ERAS principles to elective craniotomy significantly improved postoperative outcomes, including shorter hospital stays, reduced costs, fewer complications such as nausea and vomiting, and earlier patient mobilisation [14]. These findings highlight the growing applicability of ERAS protocols in optimizing recovery and enhancing perioperative care across diverse surgical specialties. Despite these advances, several critical questions remain unanswered. The combined impact of multiple pharmacogenetic variants on surgical stress response has not been comprehensively evaluated and the potential benefits of pharmacogenomic -guided perioperative management in abdominal surgery patients require further investigation [15]. Furthermore, the cost-effectiveness and practical implementation challenges of routine pharmacogenetic testing in surgical populations need to be addressed [16]. Therefore, it is of interest to conduct an integrative analysis of pharmacogenomic factors and surgical stress response in patients undergoing elective abdominal surgeries.

## Materials and Methods:

## Study design and setting:

A prospective cohort study was conducted at a tertiary care academic medical center between January 2022 and December 2023.

## Sample size calculation:

Based on a preliminary analysis, we estimated that 240 patients would provide 90% power to detect a 25% difference in postoperative complication rates between pharmacogenetic groups, with a two-sided alpha level of 0.05. This calculation assumed a baseline complication rate of 25% in the standard care group and accounted for a 10% dropout rate.

## Study population:

## Inclusion criteria: 

Adult patients (≥18 years) scheduled for elective abdominal surgeries including colectomy, gastrectomy, hepatectomy, pancreatectomy, or major gynecological procedures; American Society of Anesthesiologists (ASA) physical status I-III; ability to provide informed consent; and willingness to undergo pharmacogenetic testing.

## Exclusion criteria: 

Emergency surgery; known genetic disorders affecting drug metabolism; chronic opioid use (>3 months); active systemic infection; autoimmune disorders; immunosuppressive therapy; pregnancy; and inability to comply with study procedures

## Pharmacogenetic testing:

Preoperative blood samples (5 mL) were collected in EDTA tubes from all participants. DNA extraction was performed using a commercial kit (QIAamp DNA Blood Mini Kit, Qiagen, Germany). Genotyping was conducted using a targeted next-generation sequencing panel covering the following pharmacogenetic variants: CYP2D6 (*2, *3, *4, *5, *6, *10, *17, *41), CYP3A4 (*1B, *22), OPRM1 (A118G) and COMT (Val158Met). Patients were classified according to Clinical Pharmacogenetics Implementation Consortium (CPIC) guidelines into poor, intermediate, extensive, or ultrarapid metabolizers for CYP2D6 and CYP3A4.

## Surgical stress response assessment:

Surgical stress response was evaluated through serial measurements of biomarkers at the following time points: baseline (preoperative), 6 hours, 24 hours and 48 hours postoperatively.

## Blood samples (10 mL) were collected for analysis of:

[1] Cortisol: Measured using chemiluminescent immunoassay (Access Cortisol assay, Beckman Coulter)

[2] Interleukin-6 (IL-6): Quantified by enzyme-linked immunosorbent assay (ELISA) (Human IL-6 Quantikine ELISA Kit, R & D Systems)

[3] C-reactive protein (CRP): Measured using immunoturbidimetric assay (CRP Latex, Roche Diagnostics)

[4] Tumor necrosis factor-alpha (TNF-α): Quantified by ELISA (Human TNF-α Quantikine ELISA Kit, R & D Systems)

## Intervention:

After baseline assessments, patients were stratified into two groups:

[1] Pharmacogenomic-guided group (n=120): Perioperative medications were selected and dosed based on pharmacogenetic results according to CPIC guidelines.

[2] Standard care group (n=120): Received standard perioperative management without pharmacogenetic guidance.

Anesthesia and analgesia protocols were standardized except for pharmacogenomic-guided modifications. All patients received balanced general anesthesia with sevoflurane, fentanyl and rocuronium. Postoperative analgesia included multimodal regimens with acetaminophen, NSAIDs and opioids as needed.

## Outcome measures:

## Primary outcomes:

[1] Postoperative complication rate within 30 days (Clavien-Dindo classification)

[2] Hospital length of stay

[3] Intensive care unit (ICU) admission rate and length of stay

## Secondary outcomes:

[1] Postoperative pain scores (Visual Analog Scale, VAS) at 6, 24 and 48 hours

[2] Opioid consumption (morphine milligram equivalents, MME)

[3] Surgical stress biomarker levels

[4] Quality of recovery (QoR-15 score) at 24 and 48 hours

[5] Patient satisfaction scores (5-point Likert scale)

## Statistical analysis:

Data were analyzed using SPSS version 28.0 (IBM Corp., Armonk, NY). Continuous variables were expressed as mean ±standard deviation (SD) and compared using Student's t-test or Mann-Whitney U test as appropriate. Categorical variables were presented as frequencies and percentages and compared using chi-square or Fisher's exact tests. Repeated measures ANOVA were used to analyze changes in biomarker levels over time. Multivariate logistic regression analysis was performed to identify independent predictors of postoperative complications. A p-value <0.05 was considered statistically significant.

## Results:

A total of 240 patients were enrolled in the study, with 120 patients assigned to each group. The demographic and clinical characteristics were comparable between groups (Table 1 - see PDF). The mean age was 58.3 ±12.6 years and 52.5% were female. The most common surgical procedures were colectomy (35.8%), gastrectomy (24.2%) and hepatectomy (18.3%). The distribution of pharmacogenetic variants was similar between groups, with CYP2D6 poor metabolizers comprising 8.3% of the study population. Patients with CYP2D6 poor metabolizer status demonstrated significantly higher IL-6 levels at 24 hours postoperatively compared to extensive metabolizers (187.3 ±42.6 pg/mL vs. 142.8 ±38.2 pg/mL, p=0.003). Similarly, CYP2D6 poor metabolizers exhibited elevated TNF-α levels at 48 hours (28.7 ±6.8 pg/mL vs. 22.4 ±5.9 pg/mL, p=0.008). OPRM1 A118G variant carriers showed significantly higher cortisol levels at 6 hours postoperatively (28.4 ±7.2 µg/dL vs. 23.1 ±6.5 µg/dL, p=0.002) and increased pain scores throughout the postoperative period. The pharmacogenomic-guided group demonstrated significantly better outcomes compared to the standard care group (Table 2 - see PDF). Postoperative complication rates were 32% lower in the pharmacogenomic-guided group (18.3% vs. 26.7%, p=0.042). Hospital length of stay was reduced by 1.6 days in the pharmacogenomic-guided group (5.2 ±1.8 days vs. 6.8 ±2.1 days, p<0.001). Opioid consumption was 28% lower in the pharmacogenomic-guided group (45.2 ±12.3 MME vs. 62.8 ±15.7 MME, p<0.001). Serial measurements of surgical stress biomarkers revealed significant differences between groups over time (Table 3 - see PDF). The pharmacogenomic-guided group showed lower peak IL-6 levels (165.4 ±38.7 pg/mL vs. 198.2 ±45.3 pg/mL, p=0.001) and faster normalization of CRP levels. Cortisol levels were significantly lower in the pharmacogenomic-guided group at all postoperative time points (p<0.05 for all comparisons). Multivariate logistic regression identified CYP2D6 poor metabolizer status (OR 2.8, 95% CI 1.4-5.6, p=0.004) and OPRM1 A118G variant (OR 2.1, 95% CI 1.2-3.8, p=0.012) as independent predictors of postoperative complications. Pharmacogenomic-guided management was associated with a 45% reduction in complication risk (OR 0.55, 95% CI 0.32-0.94, p=0. 029).

## Discussion:

This study provides compelling evidence for the integration of pharmacogenomics into perioperative care for patients undergoing elective abdominal surgeries. Our findings demonstrate that pharmacogenetic profiling significantly influences surgical stress response and postoperative outcomes, with pharmacogenomic-guided management resulting in substantial clinical benefits interventions and [17]. The observed association between CYP2D6 poor metabolizer status and elevated inflammatory markers aligns with previous research highlighting the role of genetic variants in modulating immune responses [18]. Patients with CYP2D6 poor metabolizer status exhibited significantly higher IL-6 and TNF-α level, suggesting that impaired drug metabolism may contribute to an exaggerated inflammatory response. This finding is particularly relevant given the well-established relationship between excessive inflammation and postoperative complications [19]. Our results extend the current understanding of pharmacogenomics in perioperative medicine by demonstrating the clinical utility of preoperative genetic testing [20]. The 32% reduction in postoperative complications observed in the pharmacogenomic-guided group represents a significant improvement in patient outcomes, consistent with emerging evidence supporting personalized approaches to surgical care [21]. The reduction in hospital length of stay by 1.6 days has important implications for healthcare resource utilization and cost-effectiveness [22]. The relationship between OPRM1 A118G polymorphism and increased pain scores, cortisol levels and analgesic requirements corroborates previous findings regarding the influence of opioid receptor genetics on perioperative outcomes [23]. This variant, which results in reduced receptor binding affinity, appears to contribute to both heightened stress response and inadequate pain control, creating a challenging clinical scenario that requires personalized management strategies [24]. The observed differences in surgical stress biomarker trajectories between groups provide mechanistic insights into the benefits of pharmacogenomic-guided care. Lower peak IL-6 levels and faster CRP normalization in the intervention group suggest more controlled inflammatory responses, likely resulting from optimized medication selection and dosing [25]. These findings support the concept that pharmacogenomic interventions can modulate the surgical stress response at a molecular level [26]. The 28% reduction in opioid consumption in the pharmacogenomic-guided group is particularly noteworthy given the current opioid crisis and the need for strategies to minimize opioid exposure [27]. This reduction was achieved without compromising pain control, as evidenced by lower pain scores and higher patient satisfaction in the intervention group. These findings suggest that pharmacogenomic guidance can facilitate more effective and safer pain management strategies [28]. Our multivariate analysis identified CYP2D6 poor metabolizer status and OPRM1 A118G variant as independent predictors of postoperative complications, highlighting the potential value of these markers in risk stratification [29]. The identification of high-risk patients through pharmacogenetic testing could enable targeted resource allocation, potentially improving outcomes while optimizing health care efficiency [30].The strengths of our study include its prospective design, comprehensive pharmacogenetic profiling and detailed assessment of surgica stress response. However, several limitations should be acknowledged. The single-center design may limit generalizability and the relatively short follow-up period prevents assessment of long-term outcomes. Additionally, the study focused on a limited set of pharmacogenetic variants and future research should explore a broader range of genetic factors [31]. The clinical implications of our findings are substantial. The integration of pharmacogenomic testing into routine preoperative assessment could identify patients at risk for adverse outcomes and guide personalized perioperative management [32]. The observed benefits in terms of reduced complications, shorter hospital stays and decreased opioid consumption support the cost-effectiveness of this approach, particularly for high-risk surgical populations [33]. Future research should focus on validating these findings in larger, multicenter studies and exploring the long-term impact of pharmacogenomic-guided care on patient outcomes and healthcare costs [34]. Additionally, building user-friendly clinical decision support platforms that can operationalize pharmacogenomic recommendations in day-to-day prescribing remains a critical future priority, as the physiologic stress response to surgery can precipitate immune dysregulation-thereby increasing susceptibility to postoperative complications, including infection and impaired wound repair [35].

## Conclusion:

Pharmacogenomic profiling plays a pivotal role in predicting surgical stress response and tailoring perioperative care. Incorporating genetic testing into preoperative assessment enhances clinical outcomes and resource efficiency. Thus, we show the value of personalized medicine in optimizing surgical management and patient recovery.
